# Supplementation of *Lycium barbarum* Polysaccharide Combined with Aerobic Exercise Ameliorates High-Fat-Induced Nonalcoholic Steatohepatitis via AMPK/PPARα/PGC-1α Pathway

**DOI:** 10.3390/nu14153247

**Published:** 2022-08-08

**Authors:** Dou-Dou Li, Jia-Min Ma, Ming-Jing Li, Lu-Lu Gao, Yan-Na Fan, Yan-Nan Zhang, Xiu-Juan Tao, Jian-Jun Yang

**Affiliations:** 1 School of Public Health and Management, Ningxia Medical University, Yinchuan 750004, China; 2 Yuyang District Center for Disease Control and Prevention, Yulin 719000, China; 3 School of Public Health, Xinxiang Medical University, Xinxiang 453003, China; 4 Ningxia Key Laboratory of Environmental Factors and Chronic Disease Control, Yinchuan 750004, China

**Keywords:** nonalcoholic steatohepatitis, *Lycium barbarum* polysaccharide, aerobic exercise, fatty acid oxidation

## Abstract

Nonalcoholic steatohepatitis (NASH) is a subtype of nonalcoholic fatty liver disease (NAFLD). Either *Lycium barbarum* polysaccharide (LBP) or aerobic exercise (AE) has been reported to be beneficial to hepatic lipid metabolism. However, whether the combination of LBP with AE improves lipid accumulation of NASH remains unknown. Our study investigated the influence of 10 weeks of treatment of LBP, AE, and the combination (LBP plus AE) on high-fat-induced NASH in Sprague–Dawley rats. The results showed that LBP or AE reduced the severity of the NASH. LBP plus AE treatment more effectively ameliorated liver damage and lowered levels of serum lipid and inflammation. In addition, the combination can also regulate genes involved in hepatic fatty acid synthesis and oxidation. LBP plus AE activated AMPK, thereby increasing the expression of PPARα which controls hepatic fatty acid oxidation and its coactivator PGC-1α. Our study demonstrated the improvement of LBP plus AE on NASH via enhancing fatty acid oxidation (FAO) which was dependent on AMPK/PPARα/PGC-1α pathway.

## 1. Introduction

At present, nonalcoholic fatty liver disease (NAFLD) has been one of the most common causes of chronic liver diseases associated with type 2 diabetes as well as metabolic syndrome. The disease spectrum of NAFLD involves a spectrum of conditions ranging from nonalcoholic fatty liver (NAFL), nonalcoholic steatohepatitis (NASH), cirrhosis, and hepatocellular carcinoma (HCC). Nowadays, as a more aggressive form of the disease, NASH can lead to higher risk of cirrhosis, HCC, and the increased liver-related mortality in individuals with NAFLD [1]. It has been reported that NASH has been nowadays recognized as a serious health issue, affecting 3–5% of the world’s population [2,3,4]. It is estimated that 6.65 million American adults had NASH in 2017 and $222.6 billion would be spent by those people over their life span [5]. Based on a meta-analysis of paired-biopsy studies, liver fibrosis progresses more rapidly in patients with NASH than nonalcoholic fatty liver [6]. In addition, NASH is found to be the fastest-increasing indication for liver transplant in the United States in 2019 [7]. Lipid acquisition exceeds disposal, resulting in lipid deposition in the liver. There are two ways of intrahepatic lipid disposal: oxidation (in the mitochondria, peroxisomes, and cytochromes) and export of lipids in very low-density lipoproteins [8]. Basal hepatic fat oxidation was found to be reduced in overweight patients with NAFLD, indicating that enhancement of fat oxidation may be a therapeutic strategy for NAFLD [9].

As a heteropolysaccharide, *Lycium barbarum* polysaccharide (LBP), one of the main active components of *Lycium barbarum*, exhibits a range of effects of promoting health, such as lowering blood glucose [10], lowering blood lipids [10], regulating immunological activities [11], reducing oxidative stress [12] and so on. There have been some studies demonstrating that LBP is beneficial to the liver. LBP markedly decreases the accumulation of triglyceride in the liver by deacetylation of SIRT1 [13]. Using a methionine-choline deficient mouse as a NASH model, LBP was found to have hepatoprotective effects by suppressing NLRP3/6 pathway and NF-κB activation [14]. Moreover, oxidative stress, lipid accumulation, and inflammatory response in the liver were reported to be improved by LBP based on the NASH rats and cellular steatosis model [15].

Lifestyle changes (balanced diet and proper exercise) are proposed to be the primary intervention of NASH [16], among which the importance of exercise is self-evident. Based on a randomized controlled trial of 47 diabetic obese people with NAFLD, Walid Kamal Abdelbasset et al. found that intrahepatic triglycerides and visceral lipids were reduced in people doing high-intensity interval or moderate-intensity continuous aerobic exercise (AE) [17]. In addition, patients with NAFLD improved their intrahepatic triglycerides and decreased body mass index after AE training [18]. As LBP or aerobic exercise possess these protective roles against NAFLD, respectively, whether the combination of LBP and AE could play a further synergetic role in preventing NASH needs further investigation.

The AMP-activated protein kinase (AMPK), a serine/threonine protein kinase, was demonstrated to play an important part in hepatocyte fatty acid metabolism [19]. By murine and simian models, activation of AMPK was demonstrated to ameliorate the progression of NASH [20]. As ligand-activated transcription factors, peroxisome proliferator-activated receptors (PPARs) regulate genes which play vital roles in cell differentiation and various metabolic processes [21]. One of the isoforms is PPARα, which is mainly expressed in fatty tissues [22]. Hepatic PPARα was reported to be required for protection in steatohepatitis [23]. Peroxisome proliferator-activated receptor γ coactivator 1α (PGC-1α) performs a critical regulation in the expression of related genes involved in lipid metabolism [24]. It is reported that PGC-1α and PPARα can cooperate to regulate the expression of genes involved in mitochondrial fatty acid oxidation [25].

In this study, we aim to explore the effects of LBP, AE, and LBP plus AE on nonalcoholic steatohepatitis in high-fat-induced Sprague–Dawley rats and the underlying mechanism. Our results indicate that all three treatments had benefits and LBP plus AE showed the greatest benefit. LBP plus AE alleviated fat accumulation in rats with NASH by AMPK/PPARα/PGC-1α pathway.

## 2. Materials and Methods

### 2.1. Experimental Protocol

**Figure 1:**
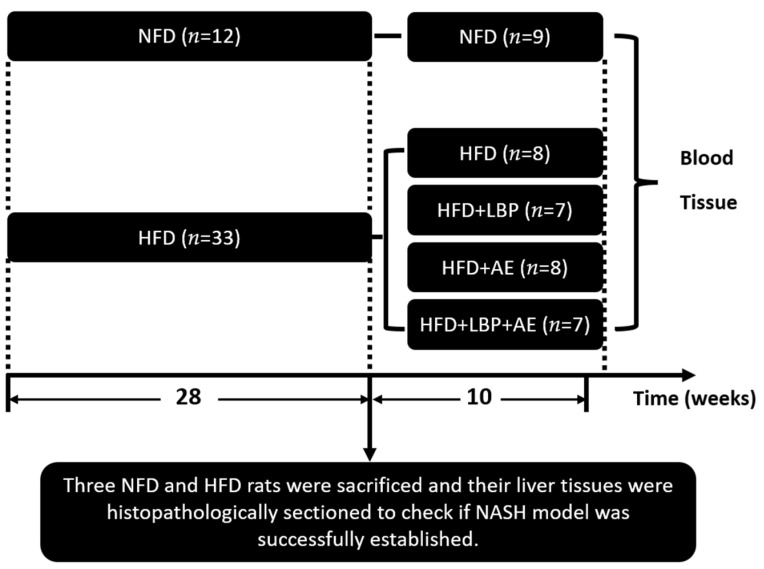
Experimental design. NFD = normal-fed diet, HFD = high-fat fed diet, LBP = *Lycium barbarum* polysaccharide, AE = aerobic exercise.

Eight-week-old male Sprague–Dawley rats (180–220 g) were obtained from the Experimental Animal Center of Ningxia Medical University (license number: SCXK Ning. 2015-0001) and maintained on a 12-h light/12-h dark cycle. All rats had free access to food and drink. Rats were randomly distributed into two groups: the normal-fat diet group (NFD) consuming the normal diet (Beijing Keao Xieli Feeds Co., Ltd., Beijing, China, *n* = 12), and the high-fat fed diet group (HFD) (60 kcal% from fat, MD12033, Medicience Ltd., Jiangsu, China, *n* = 33). Twenty-eight weeks later, three NFD and HFD rats were sacrificed, and their liver tissues were histopathologically sectioned to check if NASH models were successfully established according to Guidelines for the diagnosis and treatment of nonalcoholic fatty liver diseases [26]. Nonalcoholic fatty liver activity score (NAS) was used for evaluating liver damage. Then rats in HFD group were randomly assigned into four groups: HFD (high-fat diet) (*n* = 8), HFD plus LBP (high-fat diet with LBP supplement, 100 mg/kg) (*n* = 7), HFD plus AE (high-fat diet and aerobic exercise) (*n* = 8), HFD plus LBP plus AE (high-fat diet with supplementation of LBP combined with aerobic exercise) (*n* = 7) for 10 weeks (Figure 1).

### 2.2. Aerobic Exercise and the Source of LBP

The HFD plus AE and HFD plus LBP plus AE groups were adapted to a treadmill (Panlab/Harvard apparatus small animal treadmills, LE8710RTS, RWD Life science Co., Ltd., Shenzhen, China) for one week (speed from 5 m/min to 20 m/min, time from 10 min/day to 50 min/day). From the second week, rats did aerobic exercise at the speed of 20 m/min, 60 min/day, 5 days/week. LBP (B20460) was from Shanghai Yuanye Biological Technology Co., Ltd. (Shanghai, China). The characterization of LBP was performed in previous study [27].

### 2.3. Oil Red O Staining

Collected liver tissues stored in liquid nitrogen were embedded in optimal cutting temperature compound. Sections (4 μm thick) were stained with oil red (Nanjing Jiancheng Institute of Bio Engineering, Inc., Nanjing, China) and visualized under the microscope to determine the degree of fat accumulation.

### 2.4. Transmission Electron Microscopy (TEM)

Collected liver tissues were cut into no more than 1 mm^3^. Then they were immediately placed in 2.5% glutaraldehyde after washing in pre-cooled saline and fixed in the refrigerator at 4 °C for 4 h. The sections were fixed with osmium acid after washing with PBS three times. The washing steps were repeated, followed by ethanol gradient dehydration and epoxy resin embedding. The ultrastructure of the liver tissues was observed by TEM (HT7800, Hitachi, Tokyo, Japan) after special staining.

### 2.5. Biochemical Analyses and Enzyme-Linked Immunosorbent Assay (ELISA)

Blood samples were collected from the abdominal aorta. According to the manufacturer’s instructions, aspartate aminotransferase (AST) serum alanine aminotransferase (ALT), triglycerides (TG), and total cholesterol (TC) were measured by assay kits (Nanjing Jiancheng Institute of Bio Engineering, Inc., Nanjing, China). The levels of monocyte chemotactic protein-1 (MCP-1), Interleukin-6 (IL-6), and tumor necrosis factor-α (TNF-α) were determined by the commercial ELISA kits (Elabscience Biotechnology Co., Ltd., Wuhan, China).

### 2.6. Total RNA Isolation and Real-Time Polymerase Chain Reaction (qRT-PCR)

The messenger RNA (mRNA) expressions of toll-like receptor 4 (TLR4), mitogen-activated protein kinases 38 (p38 MAPK), and nuclear transcription factor kappa B1 (NF-ΚB 1) were determined by qRT-PCR. Total RNA extraction was prepared from liver tissue by Trizol reagent (Ambion, Austin, DX, USA) and the cDNAs were generated by reverse transcription kit (Takara, Kusatsu, Japan). A qRT-PCR was performed using a standard protocol in the real-time PCR system (Bio-Rad, Hercules, CA, USA). The primer sequences for qPCR are listed in Table 1. The expression levels of target genes were normalized to β-actin mRNA.

### 2.7. Western Blot

Three samples of mice in each group were used, from which 100 mg of liver tissue was homogenized using lysis buffer for total protein extraction and the concentration of protein was assessed by BCA protein assay kit. After being separated on sodium dodecyl sulfate-polyacrylamide gel electrophoresis (SDS-PAGE), protein samples were transferred onto polyvinylidene difluoride membranes. Membranes were blocked with 5% (*w*/*v*) skim milk for 1.5 h and then were incubated with the primary antibodies overnight at 4 °C. TLR4 (1:1000, Cell Signaling Technology, Danvers, MA, USA) and AMPK (1:1000, Cell Signaling Technology, USA), p-AMPK (1:1000, Cell Signaling Technology, USA), PPARα (1:1000, Santa Cruz Biotechnology, Dallas, DX, USA), PGC-1α (1:1000, Abcam, Cambridge, UK), and β-actin (1:5000, Abcam, UK). After 3 × 10 min wash in PBST, the secondary antibody was added and incubated for 1 h. A chemiluminescence detection was responsible for protein chemiluminescence and a bioanalytical imaging system for signal capture. Western blot was repeated three times.

### 2.8. Statistical Analysis

Using SPSS23.0 software (Chicago, IL, USA), the results were analyzed by one-way analysis of variance (ANOVA) among multiple groups and least significant difference (LSD) test for further pairwise comparison. Data are presented as mean ± standard deviation (SD) and considered significantly different as values of *p* < 0.05.

## 3. Results

### 3.1. Supplementation of LBP with AE Alleviated Hepatocyte Injury in NASH Rats

**Figure 2:**
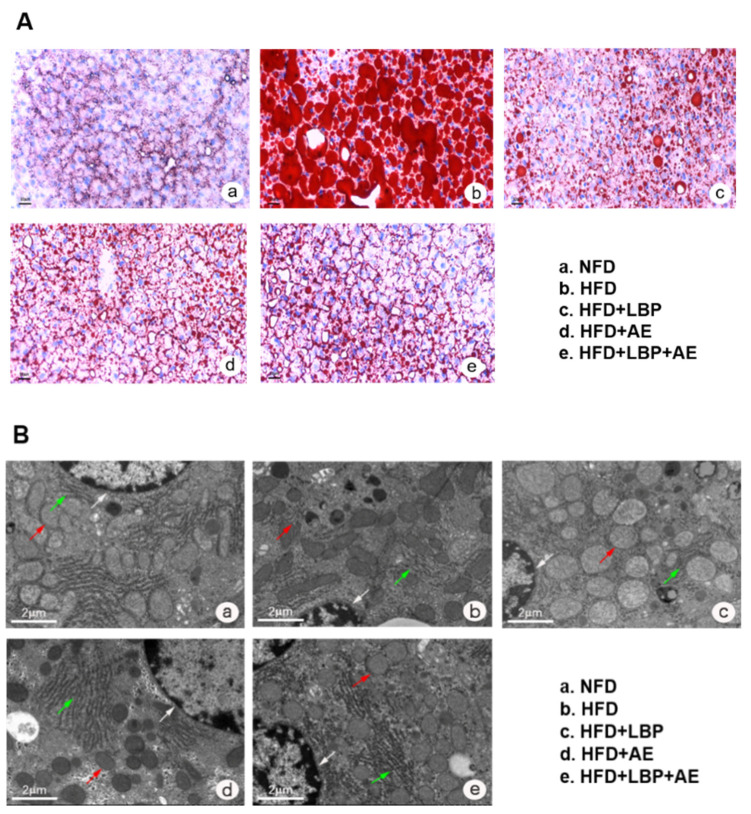

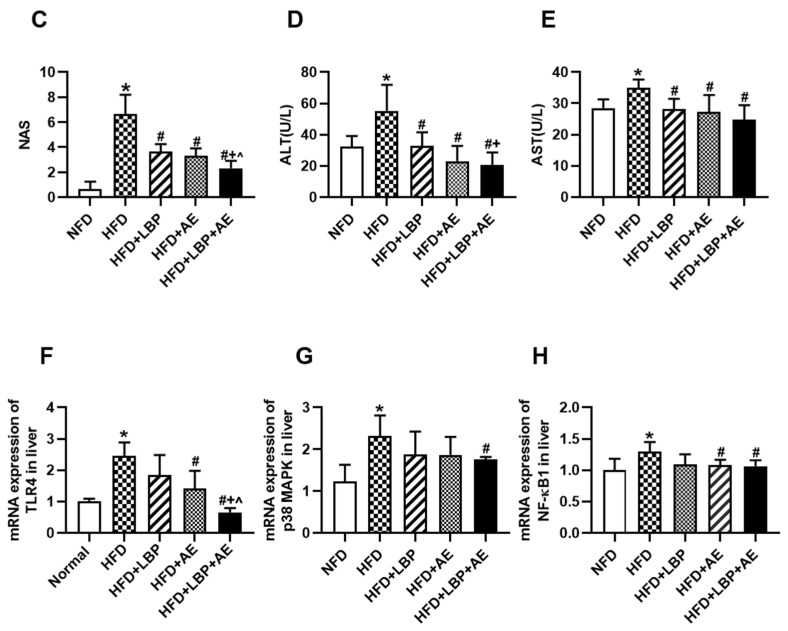
LBP plus AE ameliorated the hepatic injury. (**A**) Oil red O staining of the liver tissue was photographed at 200× magnification. (**B**) Transmission electron microscopy of the liver histology was photographed at 6000× magnification. White arrow: nucleus. Red arrow: mitochondrion. Green arrow: rough endoplasmic reticulum. (**C**) Hepatic NAS in different groups. (**D**) Serum levels of ALT. (**E**) Serum levels of AST. (**F**) mRNA expression of TLR4 in liver. (**G**) mRNA expression of p38 MAPK in liver. (**H**) mRNA expression of NF-kB1 in liver. * *p* < 0.05 vs. the NFD group. # *p* < 0.05 vs. the HFD group. + *p* < 0.05 vs. the HFD plus LBP group. ^ *p* < 0.05 vs. the HFD plus AE group.

Compared with the NFD group, HFD successfully induced severe NASH (Figure 2C). Nonalcoholic fatty liver activity score (NAS) was reduced in each treatment group to varying degrees, with the most significant reduction in HFD plus LBP plus AE group (Figure 2C). As visualized in Oil Red O staining, there were a large number of lipid vacuoles in the cytoplasm of hepatocytes in the model group (Figure 2A). Different treatments obviously reduced the degree of lipid deposition in the hepatocyte. In addition, compared with the NFD group, we found more lipid droplets, karyopyknosis, swollen mitochondria, dilation of endoplasmic reticulum, and reduction of rough endoplasmic reticulum in the HFD group (Figure 2B). However, the morphological structures of the nucleus and mitochondria were improved by all treatments. Thirty-eight weeks of high-fat diet led to an increase in ALT and AST levels by 70.23% and 23.01% compared with the NFD group, however, LBP, AE, and combination therapy groups obviously decreased the levels (*p* < 0.05). LBP plus AE was the most significant way to reduce the level of ALT statistically and AST in trend (Figure 2D,E). As NASH is reported to be an inflammatory subtype of NAFLD [2], the levels of liver inflammatory factors in HFD group were higher than that in NFD group. (Figure 2F–H). The levels of TLR4 and NF-κB1 were reduced after 10 weeks of AE with or without LBP supplementation (Figure 2F,H). Notably, AE combined with LBP was the most effective way to reduce the mRNA expression of TLR4 and p38 MAPK (Figure 2F,G).

### 3.2. Supplementation of LBP with AE Reduced Body Weight, Serum Lipid and Inflammation Levels

**Figure 3:**
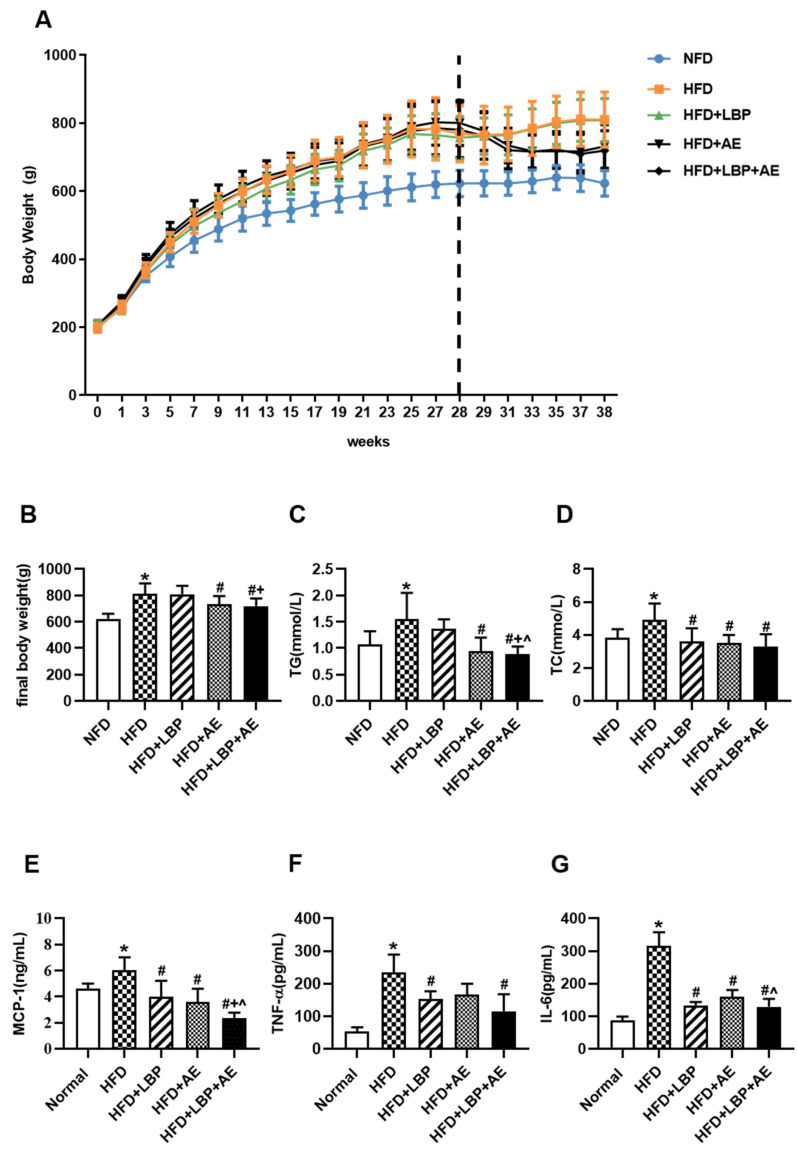
LBP plus AE reduced body weight, serum lipid as well as inflammation levels. (**A**) Body weight throughout the 38-week period. (**B**) Final body weight. (**C**) Serum levels of TG. (**D**) Serum levels of TC. (**E**) Serum levels of MCP-1. (**F**) Serum levels of TNF-α. (**G**) Serum levels of IL-6. * *p* < 0.05 vs. the NFD group. # *p* < 0.05 vs. the HFD group. + *p* < 0.05 vs. the HFD plus LBP group. ^ *p* < 0.05 vs. the HFD plus AE group.

Using a rat model of NASH, we noticed that HFD feeding for 10 weeks led to obvious increases in body weight compared with standard diet feeding (Figure 3A,B). In terms of final body weight, the HFD group increased by 30.04% compared with the NFD group. In addition, the body weight of the AE group and the LBP plus AE group decreased by 9.72% and 11.25% compared with the model group, respectively (Figure 3B). AE and LBP plus AE reduced TG level by 39.4% and 43.2%, respectively, relative to the HFD group (Figure 3C). All treatments had the capacity to lower the level of TC relative to the HFD group (Figure 3D). Then the effects of LBP, AE, and both on systemic inflammation was examined. Obviously, relative to the NFD group, higher values for inflammatory factors can be found in the HFD group. Compared with the HFD group, all treatments have reduced the level of MCP-1 by 33.6%, 40.6%, and 60.9% (Figure 3E). As shown in Figure 3F, the level of TNF-α of rats significantly decreased by supplementation of LBP (*p* < 0.05) and the combination therapy (*p* < 0.01) compared with the HFD group. In addition, all treatments can lower the level of IL-6 in comparison with the HFD group (*p* < 0.01) and LBP plus AE was more effective than AE alone (Figure 3G).

### 3.3. Supplementation of LBP with AE Regulated the Expression of Genes Involved in Lipid Metabolism

**Figure 4:**
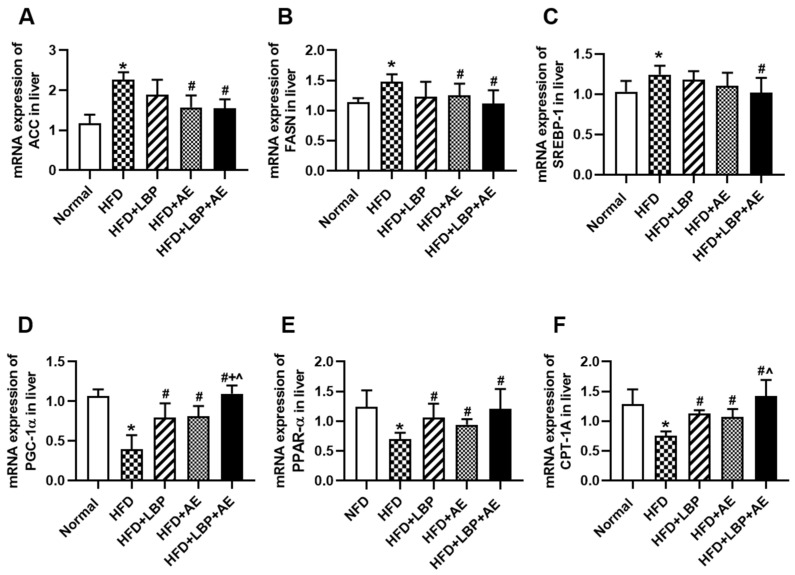
LBP plus AE affected hepatic fatty acid synthesis and oxidation. (**A**) mRNA expression of ACC in liver. (**B**) mRNA expression of FASN in liver. (**C**) mRNA expression of SREBP1c in liver. (**D**) mRNA expression of PGC-1α in liver. (**E**) mRNA expression of PPARα in liver. (**F**) mRNA expression of CPT-1A in liver. * *p* < 0.05 vs. the NFD group. # *p* < 0.05 vs. the HFD group. + *p* < 0.05 vs. the HFD plus LBP group. ^ *p* < 0.05 vs. the HFD plus AE group.

Acetyl-CoA carboxylase (ACC), fatty acid synthase (FASN) and sterol regulatory element-binding protein 1c (SREBP1c) are genes related to de novo lipogenesis (DNL). The expression levels of the three genes were obviously higher in the HFD group compared with the NFD group, suggesting that lipid synthesis in the liver was indeed increased in the presence of NASH, thereby causing fat accumulation. Compared with the HFD group, different treatments reduced the expression levels of the three genes to varying degrees. There were no differences in the expression levels of ACC and FASN between single treatment and combined treatment (Figure 4A,B). LBP and AE tended to decrease SREBP1c expression levels, but only LBP plus AE made a statistical decrease (Figure 4C). As a gene strongly associated with lipid metabolism [24], hepatic expression of PGC-1α was higher through different treatment groups in comparison with the HFD group and the combination therapy group had the most significant effect (Figure 4D). We then examined the expression of two genes of fatty acid oxidation (FAO). It turned out that treatments effectively prevented the decrease of PPARα and carnitine palmitoyltransferase-1A (CPT-1A) (Figure 4D,F).

### 3.4. Supplementation of LBP with AE Alleviated NASH via AMPK/PPARα/PGC-1α Pathway

**Figure 5:**
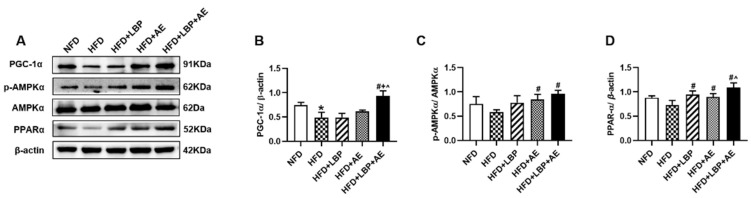
LBP plus AE activated AMPK/PPARα/PGC-1α pathway to ameliorate NASH. (**A**–**D**) Western blot images and quantitative band density analyses in different groups. * *p* < 0.05 vs. the NFD group. # *p* < 0.05 vs. the HFD group. + *p* < 0.05 vs. the HFD plus LBP group. ^ *p* < 0.05 vs. the HFD plus AE group. Three samples of mice in each group were used and western blot was repeated three times.

To further explore the effect of supplementation of LBP with AE on the AMPK/PPARα/PGC-1α pathway in NASH, proteins were extracted from the liver tissue and the expressions of AMPK, PPARα, and PGC-1α were determined (Figure 5A). HFD decreased the expression of PGC-1α (Figure 5B). Compared with the HFD group, either LBP or AE e treatment did not cause significant changes, while LBP plus AE upregulated PGC-1α at a significant level of *p* < 0.05. Rats in the AE and LBP plus AE group exerted a higher expression of *p*-AMPK (Figure 5C). In addition, all treatments upregulated the levels of PPARα, and LBP plus AE was more effective than AE alone (Figure 5D).

## 4. Discussion

**Figure 6:**
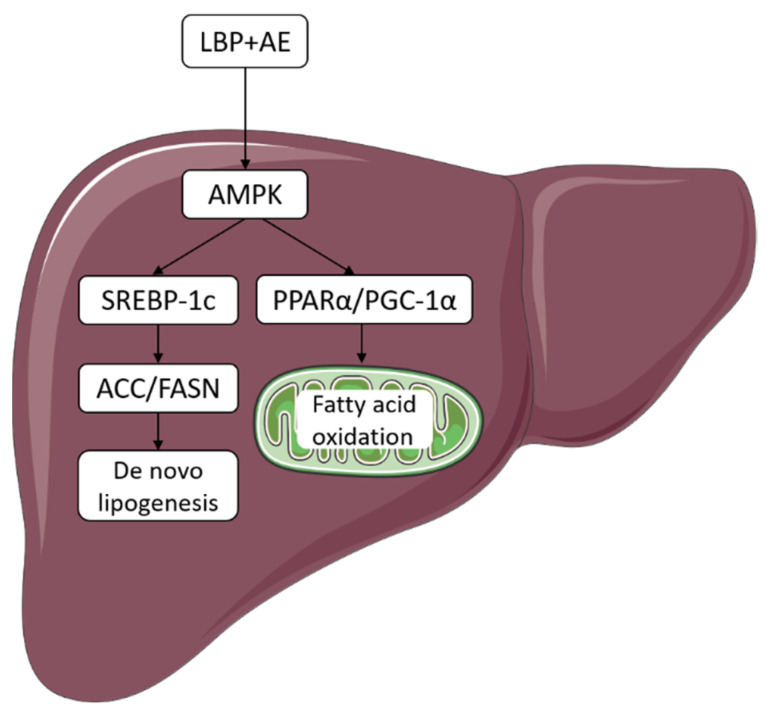
LBP plus AE improved NASH through AMPK activation and AMPK/PPARα/PGC-1α pathway. LBP plus AE activated AMPK to ameliorate NASH in two ways. On the one hand, the level of ACC and FASN decreased after SREBP-1c downregulation, thus inhibiting DNL. On the other hand, PGC-1α and PPARα can cooperate to promote FAO.

This research determined the protective effect of supplementation of LBP, AE alone, and the combination against NASH, and further revealed that the combination therapy was more effective in ameliorating NASH by inhibiting DNL and activating AMPK/PPARα/PGC-1α pathway (Figure 6).

NASH, the inflammatory subtype of NAFLD, is considered a major health issue with significant economic impact. Unfortunately, there are no pharmaceuticals for NASH that the FDA has approved in current times [28]. Vitamin E as well as pioglitazone have proved effective against NASH in randomized controlled trials [29], yet the two drugs are limited because of side effects [30,31]. Although some other drugs are in phase Ⅱ and Ⅲ trials, efficacy, safety, and other aspects still need further research [32]. Therefore, developing other new promising therapeutic strategies is still needed at present. LBP, one of the most functional components of *Lycium barbarum*, has been reported to exert benefits on NASH. Our observations that LBP produced obvious improvement in hepatic steatosis and inflammation are consistent with the results of other studies [13,14,15]. Previous studies have highlighted the positive effect of AE on people with dyslipidemia [33], which is one of the features of NASH. Our results showed that AE not only lowered TG and TC, but also improved liver function and inflammation. Then, we began to explore the synergetic effect of LBP and AE on NASH. Notably, the combined effect was better than the single effect. In addition, we also found the weight of mice in the HFD group drops in the late stage, which is consistent with the animal experiment by Eun Soo Lee et al. [34]. As for the reason, we analyze that on the one hand, it may be the abnormal liver fat metabolism in the late stage of NASH, resulting in the disorder of animal lipid metabolism, which then causes the occurrence of this phenomenon. On the other hand, changes in food intake may lead to changes in body weight, which has also appeared in our other work [35].

DNL is one method of lipid acquisition for the liver. At first, ACC catalyzes the formation of malonyl-CoA from acetyl-CoA. Then, malonyl-CoA is converted to palmitic acid by FASN. After desaturation, elongation, esterification, and other steps, triglyceride is finally formed. By isotope analysis, subjects with NAFLD are found to have high levels of DNL [36]. In an open-label study, individuals with NASH were given ACC inhibitor GS-0976 and their hepatic DNL as well as markers of liver damage were reduced after 12 weeks [37]. In another 12-week trial, 3 mg FASN inhibitor FT-4101 has been shown to reduce hepatic DNL and steatosis [38]. In our study, we found that expression of ACC and FASN declined after AE and LBP plus AE treatments. In addition, LBP plus AE was more effective than AE in trend. SREBP1c, a key transcription factor in the regulation of DNL, was reported to be an effective target to treat NAFLD after its downregulation [39]. As an upstream of SREBP1c, AMPK is able to phosphorylate SREBP-1c, which inhibits the transcription of the factor and improves hepatic steatosis [40]. Berberine and Antrodan have been demonstrated to be effective on NAFLD through AMPK-SREBP-1c pathway [41,42]. LBP has also been shown to regulate DNL by AMPK/SREBP-1c pathway [43], then we began to investigate if LBP combined with AE would also work through the pathway. Our study showed that LBP and AE alone tended to reduce the expression of SREBP-1c level in the liver with no statistical significance, while LBP plus AE significantly lowered the hepatic expression of SREBP-1c (*p* < 0.05). Therefore, LBP plus AE can activate AMPK, thereby reducing the expression of hepatic SREBP-1c levels and inhibiting DNL, thereby ameliorating NASH.

FAO is one of the ways to dispose of lipid in the liver. Hepatic FAO mainly occurs in the mitochondria and acyl-CoA relies on CPT-1A, a rate-limiting enzyme in the outer mitochondrial membrane, to enter mitochondria [44]. The previous study has shown that FAO is reduced in patients with NAFLD [9]. Therefore, augmented FAO appears to be a treatment for NAFLD. Statins prevented the progression of MCDD-induced NASH in a C57BL/6J mice model by increasing mitochondrial and peroxisomal FAO [45], meanwhile TXNIP/VDUP1 was also effective against steatohepatitis via FAO [46]. PPARα is considered as a major regulator of hepatic fatty acid metabolism [8,47]. Activating PPARα has been proved to have a therapeutic effect on the NASH not only in the animal model [48], but also in clinical trials [49]. PGC-1 family members are recognized as multifunctional transcriptional coregulators in metabolic pathways [50]. As PPARα and PGC-1α can interact to regulate FAO in mitochondrion, which plays an important role in hepatic lipid disposal [25], a recent study has found that Nuciferine was beneficial to hepatic steatosis by activating the PPARα/PGC1α pathway [51]. Being an upstream of PGC-1α [52], AMPK acts as a key regulator in homeostasis by regulating related enzymes. Cordycepin is able to improve NASH by activating AMPK [53]. The AMP-activated protein kinase (AMPK) and Sirtuin 1 (SIRT1) are partners which have similar functions in metabolism and energy balance, and both can regulate PGC-1α [54]. As LBP [13] and exercise [55] have been reported to influence SIRT1, related work about the combination therapy and SIRT1 is underway. Since AE has been shown to regulate NAFLD by AMPK-PPAR-α pathway [56] and LBP can prominently increase the expression of PGC-1α [43], we wondered if LBP combined with AE would also work through the pathway. Then we found that LBP plus AE promoted FAO via AMPK/PPARα/PGC-1α pathway.

Rather than an independent disease, NAFLD is closely related to metabolic disorders such as obesity and insulin resistance [57]. Due to the inaccurate nomenclature of NAFLD, a group of experts has suggested replacing the name with metabolic (disfunction) associated fatty liver disease (MAFLD), more accurately reflecting pathogenesis and better helping the development of treatment as well as clinical trial design [58]. Thus, metabolic health has become a key point in the therapy of NAFLD/NASH. LBP has been reported to be effective against insulin resistance by C57BL/6J mice consuming a high-fat diet as well as HepG2 cells [59], and also against obesity symptoms by altering the composition of intestinal flora as well as promoting the metabolism of SCFAs [60]. Similarly, aerobic exercise has also been demonstrated to improve metabolic abnormalities [61,62]. We previously showed that LBP plus AE reduced body weight and improved insulin resistance [27], thereby LBP plus AE is a good choice for the therapy of NAFLD/NASH.

## 5. Conclusions

In summary, our study identified effective effects of supplementation of LBP combined with AE on NASH induced by a high-fat diet in a Sprague–Dawley rat model. LBP plus AE not only promoted DNL by activating AMPK to affect the expression of related enzymes, but also enhanced FAO by AMPK/PPARα/PGC-1α pathway.

## Figures and Tables

**Table 1:** Primers of qRT-PCR.

Genes	Forward Primers	Reverse Primers
TLR4	AAGTTATTGTGGTGGTGTCTAG	GAGGTAGGTGTTTCTGCTAAG
p38MAPK NFKB1	AGAGTCTCTGTCGACCTGCT TGCATTCTGACCTTGCCTAT	CATCAGGGTCGTGGTACTGAG TCCAGTCTCCGAGTGAAGC

## Data Availability

Data is available from the corresponding authors upon reasonable request.
